# Telesimulation for medical students during the COVID-19 pandemic: experiences and student and faculty evaluation from a UK medical school

**DOI:** 10.1192/j.eurpsy.2022.2190

**Published:** 2022-09-01

**Authors:** H.T.T. Leung, J. Whall, H. Bruce, A. Ajaz, A. Korszun

**Affiliations:** 1 East London NHS Foundation Trust, Department Of Medical Education, London, United Kingdom; 2 Queen Mary University of London, Wolfson Institute Of Population Health, London, United Kingdom

**Keywords:** telesimulation, Covid-19, undergraduate medical education

## Abstract

**Introduction:**

During the COVID-19 pandemic, telesimulation became particularly important to continue the education of medical students during disrupted clerkships while maintaining social distancing.

**Objectives:**

To describe our experiences of adapting to telesimulation and evaluate this from student and faculty perspectives.

**Methods:**

The intervention was evaluated using anonymous surveys consisting of statements rated on a five-point Likert scale from strongly disagree to strongly agree and open-ended questions asking students and facilitators what went well, what they would change and why, and for any other comments.

**Results:**

Adaptations addressed the logistics of online delivery and the structure and content of scenarios. Logistical considerations included central organization of sessions to relieve pressures on clinicians. Pre-session case discussions were introduced to maximise time with simulated patients and give students space to socialise. Content was modified to ensure functionality online and reflect the context of the pandemic. A total of 278 students and 24 facilitators participated in the telesimulation sessions. 98.1% of students (*N*=109) rated the sessions as very good or good. Students benefited from practicing skills, especially clinical situations which they would rarely encounter as students, and receiving feedback. Facilitators (*N*=6) felt that students learnt both skills for online consultations and skills that can be transferred to face-to-face situations, but were ambivalent on whether students would benefit more from face-to-face sessions.

**Conclusions:**

Telesimulation is a safe and effective option that offers additional opportunities for students to develop telemedicine skills. Going forward, telesimulation should complement face-to-face delivery to develop future clinicians who are proficient in both remote and face-to-face working.

**Disclosure:**

No significant relationships.

